# Transcending Landscapes: Working Across Scales and Levels in Pastoralist Rangeland Governance

**DOI:** 10.1007/s00267-017-0870-z

**Published:** 2017-05-15

**Authors:** Lance W. Robinson, Enoch Ontiri, Tsegaye Alemu, Stephen S. Moiko

**Affiliations:** 1 International Livestock Research Institute, PO Box 30709, Nairobi, 00100 Kenya; 2 College of Development Studies, Department of Environment and Development, Addis Ababa University, Addis Ababa, Ethiopia; 3 ADIS-University of Nairobi and Nabara Consult, PO Box 248, Kiserian, 00206 Kenya

**Keywords:** Commons, Environmental governance, Landscape approaches, Pastoralism, Rangelands, Scale mismatch

## Abstract

Landscape approaches can be subjected to mistakenly targeting a single “best” level of governance, and paying too little attention to the role that cross-scale and cross-level interactions play in governance. In rangeland settings, resources, patterns of use of those resources, and the institutions for managing the resources exist at multiple levels and scales. While the scholarship on commons offers some guidance on how to conceptualize governance in rangeland landscapes, some elements of commons scholarship—notably the “design principles” for effective governance of commons—do not seem to apply neatly to governance in pastoralist rangeland settings. This paper examines three cases where attempts have been made to foster effective landscape governance in such settings to consider how the materiality of commons influences the nature of cross-scale and cross-level interactions, and how these interactions affect governance. In all three cases, although external actors seemed to work appropriately and effectively at community and landscape levels, landscape governance mechanisms have been facing great challenges arising from relationships beyond the landscape, both vertically to higher levels of decision-making and horizontally to communities normally residing in other landscapes. The cases demonstrate that fostering effective landscape-level governance cannot be accomplished only through action at the landscape level; it is a task that must be pursued at multiple levels and in relation to the connections across scales and levels. The paper suggests elements of a conceptual framework for understanding cross-level and cross-scale elements of landscape governance, and offers suggestions for governance design in pastoralist rangeland settings.

## Introduction

Among the premises on which community-based natural resource management (CBNRM) is based are the ideas that sustainable use of natural resources is possible and that local resource users are often better able to manage resources than the state or distant corporate managers (Brosius et al. 1998). Ecosystems and natural resources can, however, extend beyond the “local” or “community” level, as can the challenges for managing those ecosystems and resources, and thus approaches aimed at sustainable management of natural resources over larger areas have also been developed. Ecosystem-based management, landscape approaches, ecoregional planning, and others have emerged in a search for ways to reconcile the objectives of conservation and development (Noss 1983; Omernik 1995; Slocombe 1998; Sayer 2009). Landscape approaches, for instance, seek to simultaneously achieve social, economic, and environmental objectives within a *landscape*—a place-based social–ecological system that results from the interactions among people, land and natural resources, institutions, and values (Kozar et al. 2014; Minang et al. 2015). Landscape approaches involve applying tools, methods, concepts, and approaches in order to better understand and manage the interconnections among different land uses to achieve diverse objectives and secure benefits for diverse stakeholders (Sayer et al. 2013; Kozar et al. 2014; Minang et al. 2015).

Behind these ecosystem and landscape-based approaches is also the idea that past interventions have often focused on interventions at a too small a scale: agricultural development has tended to emphasize the farm or at most the village level (Milder et al. 2014); CBNRM and commons scholarship have tended to focus on local, often village-level, commons (Purcell and Brown 2005; Berkes 2009). Local administrative boundaries, however, seldom correspond to the challenges of biodiversity conservation or of maintaining sustainable resource-based livelihoods; hence, there is a need to develop decision-making processes and management systems which function beyond particular plots and particular resources to encompass entire landscapes. Landscape and ecosystem-based approaches aim to address *scale mismatch*, the fragmentation of decision-making and other institutional challenges that result from a jurisdictional/administrative scale not corresponding to relevant biophysical or social–ecological scales. Here, we use there term *scale* to refer to particular dimensions—spatial, jurisdictional, temporal, analytical, etc.—and *levels* as the units along a scale (Gibson et al. 2000; Cash et al. 2006). Interventions to improve governance, however, often pay little attention to *scale* and focus solely on *level*, mistakenly targeting a single, “best” level of governance that is thought to be suited for addressing some particular problem or managing some particular resource (Nagendra and Ostrom 2012). This caution applies equally well to landscape approaches, which, now that they are becoming more fashionable, run the risk of being understood as the idea that the landscape is the best level at which to address the problems of reconciling conservation and development objectives.

The literatures on environmental governance and more specifically on landscape and ecosystem-based management have yet to thoroughly analyze what kinds of factors are central to, and what kinds of interventions might be taken to develop and strengthen, landscape governance. While these literatures have identified that improving landscape governance requires working at multiple levels, not only the landscape level (Sayer et al. 2013; Kozar et al. 2014), this is an issue that is yet to be fully explored. The role that cross-scale and cross-level interactions play in landscape governance is poorly understood. This paper takes steps toward addressing this gap, focusing on landscape governance in dryland pastoralist settings. We consider the implications of three case studies, two among pastoralist communities in Kenya and one in Ethiopia, where attempts have been made to foster effective landscape governance. The case studies highlight the importance of taking into account the broader institutional environment, and social–ecological characteristics beyond and at levels higher than the landscape level. We conclude that fostering effective landscape-level governance is a task that must be pursued at multiple levels and in relation to the connections across scales and levels, and we offer suggestions as how this might be done in pastoralist settings. We suggest that adaptive co-management offers insights for governance design in these kinds of dryland pastoralist settings.

### Landscape Governance in Pastoralist Settings

The term *governance* denotes something different than *management*, relating to how power is exercised, who decides, and how decisions are made (Graham et al. 2003; World Resources Institute et al. 2003). *Landscape governance* refers to the organizations, institutions, and the relationships among them in a place-based spatial unit, the ways in which governance powers are distributed within and beyond this landscape, and the ways in which these governance powers are exercised. The scholarship on commons offers some guidance on how to conceptualize governance in rangeland landscapes. Rangelands are common pool resources—that is to say, they are resource systems, regardless of the property rights regime in place, for which (i) exclusion of beneficiaries from the resource is especially costly, and (ii) exploitation of the resource by one user reduces its availability for others (Ostrom et al. 1999). Commons scholarship has identified four broad types of property rights which may exist in relation to common pool resources—private property, state property, non-property or *open access*, and group property or *commons*. It has paid particular attention, obviously, to the fourth category, but also to how commons interact with the other three tenure types. For many years, much of the attention of commons researchers was directed toward local-level natural resource commons, using case studies to document and analyze examples of well-functioning commons and of ways in which commons are often undermined by factors such as inappropriate government policies. While the local may have been privileged in commons scholarship, there has been a growing recognition that local-level commons are embedded in a multi-level world (Berkes 2009).

Institutional interactions across scales and levels have been identified as a playing a critical role in addressing governance challenges which arise from broader social–ecological environments (Folke et al. 2005; Robinson and Berkes 2011). Systems of adaptive governance tend to be polycentric, having nested, quasi-autonomous decision-making units operating at different levels (Folke et al. 2005). Describing systems has having a multiplicity of independent decision centers—as being polycentric—is only meaningful and can only result in flexible self-organization if operating under a set of overarching rules (Aligica and Tarko 2012). Having rules that enable spontaneous self-organization, however, is a necessary but not sufficient condition for adaptive, polycentric governance—institutional linkages across levels are also needed. These are often facilitated by organizations that play a bridging role between local communities and organizations at other levels (Folke et al. 2005).

Pastoralist rangeland landscapes are an excellent example of the need to think, and work across and beyond particular levels. Rangeland resources exist, both ecologically and in the minds of resource users, at multiple levels: local enclosures used for milk herds; fields that each have different mixes of grass, forb, and other species; pastures made up of a number of different fields, the various pastures being used in different seasons and for different livestock species; the overall ranges within which these pastures, fields, and enclosures exist; large landscapes made up of multiple ranges; livestock routes for daily movement between pasture and water, for seasonal movement to different ranges, and for movement to markets, etc. The institutions for managing these resources can also exist at multiple levels and multiple scales. In many cases, pastoralist rangelands constitute commons. More often, however, they *contain* commons, along with other tenure types, nested within larger multifunctional, diversely governed landscapes.

Scholarship on commons has also produced conceptual frameworks which can help with the analysis and ultimately the *design* of governance systems. Action situations related to the governance of commons can be understood as relating to decisions at three different conceptual levels. The operational level relates to rules and decisions around rewards and sanctions, when, where, and how to do something, how the monitoring of actions should be done, and what information can be shared; the collective choice level relates to rules and decisions about how operational rules can be changed, and who can participate in these decisions; and the constitutional level relates to rules and decisions about how collective choice rules can be changed, and who can participate in these decisions (Ostrom 1990). It must be noted that these “levels” of decision-making are distinct from spatial and jurisdictional scales—actors at all jurisdictional levels from households to international organizations will have decisions to make at all three conceptual levels (Gibson et al. 2000).

This analysis of the nesting of rules has not received as much attention as another development in commons scholarship: the “design principles” for effective governance of commons (Ostrom 1990; Dietz et al. 2003). While there have been some additions and revisions to these principles in other publications, Ostrom’s original (1990) list of eight principles is still the most commonly referenced. Effective commons governance systems are said to be characterized by the following design principles: well-defined group and resource boundaries, congruence between appropriation and provision rules and local conditions, collective choice arrangements, monitoring, graduated sanctions, conflict resolution mechanisms, minimum recognition of rights by government authorities, and nested enterprises.

In recent years in parts of Africa, there has been a trend toward establishing, or re-establishing pastoral commons. Interventions by governments and by conservation NGOs, recognizing that mobility is a key feature of dryland pastoralism, have often targeted their support at establishment of pastoral commons across expansive landscapes, often comprised of multiple communities. Nevertheless, except for the larger geographic extent of the territory to be governed, these efforts often follow a typical CBNRM blueprint, and in some cases are directly inspired by Ostrom’s design principles (Jones 2010; Bollig and Lesorogol 2016). There are questions, however, around how well the Ostrom design principles apply in pastoralist settings. In drylands, the spatial and temporal variability of rainfall, and hence pasture resources, can be high. The ecosystems in which dryland pastoralism takes place are often governed by non-equilibrium dynamics. That is to say, ecosystem dynamics are driven by the external factor of rainfall variability rather than by internal equilibrium between forage and grazing pressure (Ellis and Swift 1988). The practice of pastoralism is adapted to these kinds of dynamics; its linchpin is the ability to respond to variability in resources by moving with livestock to those areas where rain has fallen and forage is available. The customary institutions which pastoralists have for governing common pool resources have been similarly adapted to this variability, emphasizing access to resources rather than their ownership and management (Robinson 2009). In such settings, the design principle that calls for clearly defined resource and membership boundaries does not seem to apply, at least not in a straightforward way (Niamir-Fuller 1999; Moritz et al. 2013).

It has been recognized that different combinations of design principles may be differently suited to different types of resources (Baggio et al. 2016; Schlager 2016). Generally, however, commons scholarship, including the literature on the design principles, has paid insufficient attention to the diversity of physical environments and to the materiality of commons (Agrawal 2003; Bollig and Lesorogol 2016). Effective commons institutions are not independent of the concrete material characteristics of the resources. Bollig and Lesorogol (2016) have explored some aspects of the materiality of pastoral commons, but a conceptual framework for making sense of how social institutions and material resource characteristics interact in pastoralist rangeland governance is lacking. The question of whether and how to apply the first Ostrom design principle is a case in point. The purported need for clear boundaries is confounded by the nested, multi-level nature of resources in pastoralist settings: how are resource and membership boundaries to be set when a patch of grass may be a resource used on a daily basis by one user, on an only seasonal basis by another, and even less often by some others?; how are boundaries to be drawn when the geographic extent of the common pool resources used by each user differs and in some cases no border is possible which does not bisect the resources used or claimed by at least some users? The challenge of ensuring security of resource tenure, for which clearly defined resource and group boundaries would seem to be important, without unduly restricting mobility and access to resources beyond boundaries, has proven difficult. The difficulty of striking this balance has been called “the paradox of pastoral land tenure”: “How to define spatial and social boundaries around resources and user groups in situations where spatial and social flexibility are intrinsic and essential characteristics of resource use patterns?” (Fernández-Giménez 2002: 50). In dryland pastoralist settings, where the spatial extent of resource use is often vast, landscape approaches may be a more appropriate kind of intervention than strategies which focus solely on supporting local level commons. The task of how to support effective governance in pastoralist landscapes, however, is a challenging one, one that may need to be guided by a slightly different set of principles than those which apply to local commons. Among the challenges is how to understand, and work in the context of, interactions across scales and levels that affect landscape governance.

## Methods and Study Sites

### Study Sites

This paper brings together three case studies of community-based rangeland management initiatives from the drylands of Kenya and Ethiopia. At all three sites, in the past, landscape management naturally emerged as communities adapted to natural livelihood and production exigencies related to the spatial and temporal variability in resources, but has more recently faced challenges. The gradual strengthening of the state, formal education, and the development of alternative livelihoods are among factors that have resulted in some degree of erosion of traditional governance and management systems. Meanwhile, a growing human population and climate change have contributed to degradation. These challenges have provided the motivation for external development actors—NGOs primarily, but also research institutes and government—to intervene to strengthen natural resource management and governance. Each case involved an initiative or set of initiatives undertaken by local pastoralist communities in collaboration with one or more external actors (see Table 1). Each of these initiatives involved creating and/or strengthening governance mechanisms for the rangeland landscape and attempting to reinvigorate aspects of traditional management, with the interventions aimed primarily at this level rather than at lower—e.g., village—levels. The study sites included Il’Ngwesi group ranch and conservancy and Garba Tula rangeland in Kenya, and the Gomole rangeland unit in Ethiopia. The cases were selected to represent some of the range of variation in biophysical and socio-institutional context and in approaches to fostering landscape governance in pastoralist settings.

**Table 1:** Summary of study sites

	Garba Tula	Gomole	Il’Ngwesi
Location	Isiolo County, Kenya	Yabello and Arero Woredas, Ethiopia	Laikipia County, Kenya
Area	981,900 ha	695,300 ha	9296 ha
Ethnic makeup	Primarily Borana. Significant minority populations include Somali, Turkana and Gabra	Primarily Borana. Significant minority populations include Gabra and Guji	Maasai
External actors	IUCN, RAP, IIED	PRIME project (MercyCorps, CARE, SOS Sahel)	LWF, NRT, and others

The traditional governance system of Borana pastoralists of southern Ethiopia and northern Kenya divided the land into a number of rangeland territories called *dheedas*, one of which constitutes our first case study, Garba Tula. The dheeda approximately corresponds to the administrative boundaries of Garba Tula sub-county in Isiolo County, Kenya. Garba Tula was a dry season refuge for the Borana even before some moved from Ethiopia to settle in the area permanently. In Garba Tula, the deep wells for which the dheeda was named are no longer there and the few rivers that exist are dry most of the year. The annual rainfall ranges from 150–250 mm in the north to 300–350 mm in the south (IUCN 2011). The area borders important biodiversity areas in Kenya, including Meru and Kora National Parks. Based on the provisions of Kenya’s 2010 constitution and 2016 Community Land Act, the land falls under the tenure category of “Community Land”.

Gomole, located on the Borana Plateau in southern Ethiopia, is also a dheeda, but in this case, however, the dheeda does not correspond to any administrative territories but instead straddles two woredas (administrative districts). The word *Gomole* means “better off”, a description that alludes to its diverse vegetation cover and the good quality of the pastures relative to neighboring dheedas. The altitude ranges from 1200 m, 1400 m, and 1900 m above sea level in the west, central, and eastern sections, respectively. Aridity increases moving from east to the west with an annual average rainfall of 697 mm (Lasage et al. 2010). This variation results in a very heterogeneous landscape.

The third case study is Il’Ngwesi conservancy and group ranch. *Il’Ngwesi* in the Maa language means “a place of wildlife”. It is located in the northern lowlands of Laikipia County within the expansive North Rift region of Kenya. The mean annual rainfall is 517 mm (Chege et al. 2007) and the landscape is largely semi-arid savannah grasslands mixed with shrub-lands and acacia woodlands. The ecosystem provides habitats for many species of megafauna, including African elephants (*Loxodanta africana*) and the African lion (*Panthera leo*). Wildlife conservation and livestock husbandry are closely interlinked in natural resource management strategies. On the south of the Il’Ngwesi landscape is a high altitude, more arable land adjoining the slopes of Mt. Kenya.

### Methods

The primary data gathering methods used were key informant interviews and focus group discussions (see Table 2). The information generated was used to characterize the approaches and strategies used for community mobilization and governance, and how governance for each rangeland landscape was structured, as well as to provide insights on challenges that were being faced. Interview respondents and participants in focus group discussions were selected based on location of residence within the landscape, economic activity, gender and wealth status. Some additional information was gathered at community workshops. Other methods used to gain a deeper understanding of resource use, livestock movement, and issues and challenges related to rangeland management included transect walks and spatial calendars.

**Table 2:** Interviews and focus group discussions

	Il’Ngwesi	Garba Tula	Gomole
Key informant interviews	12	24	17
Focus group discussions	3	18	8

Part of the analysis was based on a protocol that was developed by the research team to provide a structured characterization of governance and planning approaches in each case. Variables in this characterization protocol were generally straightforward and factual and obtainable through a review of documentation and a small number of interviews with key informants. These variables included the approach to defining/delineating the landscape, criteria used for its definition, identification of the key landscape-level governance organizations and institutions, the actors involved in those organizations and institutions, the authority and types of governance powers^1^ accorded to the landscape-level governance mechanisms, the way participation and representation were structured, the approach to planning at different levels, and the nature and extent of involvement of women and minorities (see Table 3). This analysis involved understanding how the landscape institutions came into being, how management plans were designed and institutionalized, and how authority was assigned and utilized in resource management. More in-depth exploration of the challenges being faced was based on focus group discussions and further interviews. Qualitative analysis of these involved identification of recurring narratives and themes. For this exploratory research, more in-depth information was collected for the Garba Tula and Gomole cases than for Il’Ngwesi. However, the authors have a longer history of interaction with Il’Ngwesi, which contributed to our understanding of ongoing institutional dynamics at there.

**Table 3:** Summary of governance and planning characteristics for each case

Characteristics	Garba Tula	Gomole	Il’Ngwesi
Definition of the landscape	Predefined	Predefined	Predefined
Criteria for definition	Traditional territory (dheeda)/administrative unit (sub-county)	Traditional territory (dheeda)	Traditional territory (several I’nkutot joined together)
Landscape-level governance mechanism(s)	Dheeda council	Rangeland council	Highest authority is the Group Ranch Committee. Below this are the ICT, and the Il’Ngwesi Company Ltd.
Authority and governance powers possessed by the landscape governance mechanism(s)	Distribution of authority is unclear and contested	Advisory role only	Full tenure, decision-making and implementation powers
Governance by whom	Communities	Communities	Communities
Form of participation and representation	Representation by communities (wards within the sub-county)	Representation by communities (Pastoralist Associations within the rangeland unit)	Representation by communities (localities within the group ranch)
Multi-level planning approach	Planning done at landscape level and lower levels is integrated in an ad hoc way	Planning mostly done above and below landscape level	Land use planning done at the group ranch level; monitoring carried out at village level
Involvement of women	No women or other ethnic groups on dheeda council	Women have direct representation on the Rangeland Council	Special seats provided for women’s representation in key group ranch organs

## Findings

Table 3 summarizes the descriptive findings used to characterize the nature of governance and planning for each of the three rangeland landscapes, based on the protocol referred to above.

### Garba Tula

The Borana community has had a traditional resource management structure embedded in the council of elders, the *Jaarsa Dheeda* or dheeda council. This resource management system, although recognized and appreciated by the colonial Kenya government, became less functional in post-independence Kenya. Respondents for this research identified this as a key factor contributing to land degradation over the years due to poor decisions regarding resource allocation, grazing routes, and use of and access to key land resources. Comments made by an elder from Garba Tula are illustrative:

They felt that in the previous years especially after independence, people have shunned the customary ways and are just freely grazing anywhere. The customary ways that were very strong in the colonial government time demarcated areas well and people were following according to the dheeda council and paramount chiefs because they had a lot of power and you couldn’t graze in an area where the community does not allow you. The Borana fully handed all issues to do with the grazing areas.

IUCN, working with a local NGO, Resource Advocacy Program (RAP), between 2009 and 2011, put in place a participatory process to re-invigorate the *dheeda council*. They facilitated the re-institutionalization of the council by the residents, and formalization of rules around grazing through which an attempt has been made to reinstate the traditional management system with a clear distinction made between wet and dry season pastures. Through RAP, the rules were presented to the Isiolo County government so that they could be incorporated into the county resource management strategy. The International Institute for Environment and Development (IIED) also facilitated a community resource mapping process as a way of strengthening the natural resource governance. The national government, represented by the National Government Administration Office (NGAO—formerly known as the Provincial Administration), the Kenya Wildlife Services (KWS) and Kenya Forests Services (KFS), and the County government have also had some influence on resource use in the region. However, authority over resources remains poorly defined. The legitimacy of the governance and resource management system put in place is questioned by both government officials and pastoralists from other places. Some of our respondents also referred to an apparent contest between national government institutions and the customary institutions. In particular, KWS operates Bissanad National Reserve, which is adjacent to Garba Tula. However, KWS also has the mandate for protection of all wildlife in the country, not only within but also beyond the protected areas. Wildlife move between the Reserve and the areas under the dheeda council, and decisions made by KWS affect and are also affected by the actions of the dheeda council.

### Gomole

The Pastoralist Areas Resilience Improvement through Market Expansion (PRIME) project is a USAID-funded initiative in Ethiopia which began in 2012 and is being led by the NGO Mercy Corps. Included within the project is a component on natural resource management, which, in our study area in Yabello and Arero Woredas in Borena Zone in southern Ethiopia, was being implemented by the NGOs CARE and SOS Sahel. PRIME’s strategy for natural resource management has been to work with customary pastoralist institutions and with rangeland units based on customary rangeland territories. In the Borana territories where they operate, this has meant that there are five rangeland units based on the five Borana dheedas in Ethiopia. Gomole, which straddles Yabello and Arero Woredas, is one of those dheedas, and at the time of the study was the dheeda where project activities had proceeded the furthest. PRIME facilitated the creation of a rangeland council which is the primary governance mechanism for the dheeda. It is made up of community representatives from across the dheeda, with the *Abba Dheeda* (lit. “the father of the range”, a customary position whose holder traditionally had responsibility for management of pasture resources) playing a prominent role. There are also structures at lower levels: the *peasant/pastoralist association* (PA), which is the lowest level government administrative unit, and the *reera*, which is a customary territory within a dheeda. Women are represented at all levels. The intention has been to create a multi-level planning process involving identification of distinct rainy season and dry season grazing areas, planning for community enclosures, siting of water points and so on, with plans at lower levels feeding into planning done at the dheeda level. These mechanisms, however, are still in their infancy. Until now, there have been challenges of receiving formal government recognition, and as a result important planning for natural resources takes place through a distinct, government controlled planning process with most of the key decisions being made at levels lower (PA) and higher (Zone) than the landscape. Several of our respondents identified the disconnect between decision-making processes created by the government on the one hand and customary forms of decision making and the dheeda structure which PRIME is working with on the other as the primary obstacle to achieving effective rangeland management. For instance, according to one elder, “Over the last decade new structures have emerged resulting in overlapping institutions and decision making mechanisms. This has meant resource management decisions are often made independently at the different levels without a joint consideration of land users’ interests and land use potential in Gomole.”

### Il’Ngwesi

Il’Ngwesi group ranch was one of the many hybrid private-communal property regimes established in Kenya’s pastoral lands in the 1960s and ‘70s as frameworks of instituting development and improved rangeland management practices in the hitherto customary tenure rangelands. Resident owners were registered as collective private owners of the new communal ranches and mandated with their management. Later, reacting to challenges of population growth, constricting pasture lands and livelihood demands posed by lifestyle changes, and to opportunities for income generation through ecotourism, Il’Ngwesi residents, with the support of external NGOs, reorganized themselves. They devised a multi-level resource governance system which involves some elements of grazing planning at neighborhood (village forum) level, group-ranch level, and when under-stress beyond group ranch boundaries. The group ranch also became organized as a community conservancy with three key community organizations at that level: a Group Ranch Committee, which is the main decision-making organ, a Community Trust that is responsible for grazing management and monitoring, and the Il’Ngwesi Company that deals with fundraising and tourism management. In addition, there are Grazing Forums for each village, which are responsible for devising and monitoring grazing arrangements at village levels. Each of these organs has seats reserved for women. There is also an executive secretariat that is in charge of implementation and coordination.

The Il’Ngwesi group ranch has a vigorous strategy for improved landscape level natural resources management. The community trust organ works with the community members to design livestock grazing patterns depending on seasonal trends of pasture and water availability. They have zoned their pasture areas into dry and wet season grazing areas, the core conservation and buffer zones. With the support of the Laikipia Wildlife Forum (LWF) and the Northern Rangelands Trust (NRT), they have initiated various activities for rangeland rehabilitation. They have re-seeded about 20 ha of degraded land with perennial grasses and implemented bunched livestock grazing. We were informed that they have also purchased land outside the group ranch to strategically establish their settlements away from the grazing areas, and thereby free up land in the group ranch for livestock and wildlife conservation. Around Mt. Kenya and the Aberdare mountains, they have purchased pieces of land which they use as holding grounds for livestock and for easy access of the pastures in the mountain forests during the very dry seasons.

Group ranch leaders told us that they have recently found it almost impossible to prevent herders from other places grazing in their territory. In 2015, Il’Ngwesi experienced an influx of large numbers of livestock, during dry spells, from pastoral communities from elsewhere in northern Kenya, and pastures which the community had set aside for recuperation and dry season grazing were heavily grazed. Armed clashes ensued between local residents and in-migrating herders and in one incident more than ten of the latter were killed. Respondents indicated that their inability to control this influx and to fully keep out non-members from partaking in the group’s communal pastures has jeopardized their efforts to institute sustainable and functional rangeland resource management practices.

### Community Engagement, Participation, and the Organization of Landscape Governance

In each of these three cases, participatory resource mapping, other participatory approaches, the strengthening of community landscape level decision-making, and extensive community consultation were key aspects of the strategy pursued by external actors (see Table 4). For example, one elder from Kinna in Garba Tula stated that, “This whole idea came because of consultation of the community.” In all three cases moreover, local communities had a pre-existing social landscape with corresponding management practices and institutions. These landscapes were socially defined by historical circumstances, the availability of resources in relation with livestock movement, and cultural affinity and sense of place. Insofar as customary institutions were adapted to local ecologies and the needs of livelihoods based on mobile livestock keeping, each of the three landscapes could be said to be both socially and ecologically defined. While in all three cases, customary institutions had, over the years, eroded to some extent, external agents who came in to work with local communities to strengthen landscape governance and resource management found it appropriate to work on the basis of these landscapes rather than delineating new territories such as on the basis of their own interests in biodiversity conservation or through some process of negotiation. In the Garba Tula and Il’Ngwesi cases, the boundaries of these landscapes were essentially identical to some formally recognized boundary: the sub-county in the case of Garba Tula and the group ranch in the case of Il’Ngwesi. For Gomole, however, the landscape did not correspond to any administrative boundary, but rather straddled two woredas (districts). NGO staff working on the PRIME project indicated that according to their assessment, customary Borana institutions are still important in decision making and, moreover, the dheeda territory is one that makes sense in ecological and livestock production terms, and so it seemed wise to work on the basis of these dheedas. In all three cases, the external organizations have worked with what existed on the ground rather than renegotiating or otherwise redefining the landscape.

**Table 4:** Summary of the approach and governance for each case

Approach and Governance Characteristics	Garba Tula	Gomole	Il’Ngwesi
Territorial approach	• Worked with pre-existing landscape (the dheeda)	• Worked with pre-existing landscape (the dheeda)	• Worked with pre-existing landscape (the group ranch), which itself had formalized a grouping of traditional I’nkutot territories
	• The dheeda essentially corresponds with the sub-county	• The dheeda does not correspond with administrative boundaries; it straddles two woredas (districts)	
Other key elements of the approach	• Revival and adaptation of dheeda institutions, especially dheeda council and traditional management practices	• Adaptation of dheeda institutions	• Assisting the group ranch to adapt its organizational structure, creating village forums at lower levels and additional structures at group ranch level
		• Involved the Abba Dheeda and other elders	• Assisting the group ranch to engage with higher levels such as through the broad network of conservancies facilitated by NRT
	• Interventions included participatory rapid appraisal and participatory mapping	• Built on the deliberative decision-making processes that characterize customary Borana governance	• Strategy included the developing an income stream from wildlife tourism
		• Interventions included participatory resource mapping and extensive community dialog	
Structure of landscape governance mechanisms	• Integration of traditional structures—the Jaarsa Dheeda (dheeda council)—and modern administrative structures	• Creation of a new organization—the rangeland council—for the traditional territory (the dheeda)	• Landscape governance based on the group ranch land holding framework
		• The Abba Dheeda was also integrated into the decision-making	• The group ranch committee oversees the ICT and the Il’Ngwesi Company Ltd., and the group ranch secretariat, which is headed by a group ranch manager and several staff members below him
Distribution of authority	• Authority is spread across multiple actors	• Authority of community and traditional governance actors—e.g., the rangeland council and the Abba Dheeda—is informal and limited	• De jure authority is clear, deriving from the Land (Group Representatives) Act (1968)
	• Government actors: KFS, KWS, NGAO, and County Government	• De jure authority lies with state institutions at zonal, woreda and PA levels, including particularly the Pastoral Development Office	• However, enforcement of group ranch property rights is incomplete
	• Some de facto authority still resides with traditional institutions	• Primary linkage from communities to the government decision-making structure is through PA leaders	
	• Grazing patterns follow provisions made by the dheeda council	• Essentially, however, the rangeland council sits outside the government decision-making structure	
	• Authority is unclear for many issues		

The integration of the traditional systems of management and governance is most obvious for Gomole and Garba Tula. In the case of Il’Ngwesi, traditional institutions are less prominent, but decision-making processes within the group ranch are nevertheless negotiated, bottom-up, and flexible. Decisions on governing the resources, allocating responsibilities and sharing of benefits at Il’Ngwesi are made collectively, some decisions being made at the group ranch level and some more day to day decisions on grazing at the village forum level. Resolutions about issues and planning are usually done at the village level, which is the smallest unit, in collaboration with the relevant committee at group ranch level. The highest authority is with the legally constituted and mandated group ranch committee. Below this, there are two independent committees: the Il’Ngwesi Community Trust (ICT) and the Il’Ngwesi Company Limited. Where necessary, issues from the village level are taken up by the group ranch committee from where they may move to a joint committee of representatives from the three main committees. The community is often called upon to ratify what this joint committee has decided on. Lines of authority, reporting, and decision-making are clear.

Whereas at Il’Ngwesi ownership of the land and the distribution of governance powers relevant for managing natural resources are well-defined and relatively straightforward, in both the Gomole and Garba Tula cases, there are multiple governance actors that have authority over the use of resources. In the case of Garba Tula, de facto rights of access to and use of the resources are significantly influenced by the customary governance system, as are the selection of representatives to sit on the Jaarsa Dheeda:

What helped out was the nomination where each area was given the number of people to nominate, then they were brought to a panel and evaluated on their stands, involvement in public initiatives in the areas, knowledge, honesty, integrity and if you can develop a proposal though that was not a core. In the traditional way there are no elections but selection on consensus (source: an officer from the NGO, RAP).

Grazing patterns follow the provisions of the dheeda council, although the council’s ability to enforce grazing patterns is limited, particularly for herders who do not reside in the area. When there is a conflict on the access and use of resources, the council of elders will refer to the central or county government. In Gomole, governance actors who have influence over management of the grazing and rangeland resources include the rangeland council, the Abba Dheeda, and government organizations at zonal, woreda and PA levels. For the most part, governance powers lie within the government administrative structures, especially the woreda which governs resources through the Pastoral Development Office.

### Challenges to Governance From Vertical and Horizontal Relationships Beyond the Landscape

In all three cases, the primary challenges to effective governance relate not to internal dynamics but rather to how governance and management are affected by communities, organizations, and institutions from beyond the landscape. Distribution of de jure tenure and governance powers varies across the three cases. In the case of Il’Ngwesi, the tenure and the rights of the community’s institutions to manage the landscape and to make and implement rules for it is, in theory, clear, deriving from its authority from the Land (Group Representatives) Act (1968). In the cases of Garba Tula and Gomole, the landscape level councils have not received formal recognition from government. What is commons in all three cases is that the landscape governance systems face challenges in exercising de facto authority.

For Gomole, the system of territories and accompanying decision-making processes and institutions is important. That customary scale is made up of territories—from smallest to largest—at ollaa, ardha, reera, madda, dheeda, and Laff Bona levels. The rangeland councils for Gomole and other dheedas are not yet fully recognized by government and therefore, while they engage in planning for pasture management across a geographic extent that makes sense in terms of herd movements and the needs of pastoralists for resource access, their ability to implement and enforce those plans is limited. As a result their role is, in practical effect, is an advisory one. In the Ethiopian administrative system, woredas, and below them kebeles (the local jurisdiction governed by a PA), are the predominant administrative units at local levels. According to a focus group discussion with Borana elders, today the PA leader represents the formal government at community level and holds the power of decision-making at the grassroots. The PA leader is, moreover, a member of the water and pasture management committee at ardha level, ardhas being relatively small traditional units but typically larger than a kebele, as well as member of the newly instituted committee at kebele level, and has a strong influence over decisions at both levels. This new, government-established water and pasture management committee at PA level—known locally known as *Koree Dheeda*, even though it operates at much smaller scale than the dheeda—is exclusively made up of PA leader, Security and Admin officer, PA manager, and Development Agent, and has sidelined elders at the local level. The PA leader and the Abba Gare—a newly established position between the ollaa (individual camp/hamlet) and reera levels—are important government agents whose accountability is upward to the woreda and who mobilize local communities in implementing what is planned at the woreda and region levels, especially on natural resources management.

The fact that Gomole straddles two woredas further complicates efforts toward establishing the legitimacy of the rangeland council. The comment of one of our respondents—a woreda government officer—is illustrative. He dryly pointed out that “in the government system there is nothing called ‘Gomole’ ”. In other words, in the government system, planning takes place within a jurisdictional scale made up of PA, woreda, zonal, and regional levels, which is different from the customary scale made up of territories at ardha, reera, madda, dheeda, and Laff Bona levels. Within the government’s jurisdictional scale, planning takes place at the PA level, which is relatively small, at the Woreda level, which is in terms of geographic extent similar to a dheeda but with completely different boundaries, and at a higher, Regional level. Within the government system, relationships are hierarchical and for the most part the key governance powers sit ultimately at regional level. The rangeland council has not found a place within these relationships, and as such is yet to become a mechanism that has a strong role in landscape governance. While it still remains important in enforcing seasonal herd movement, this has been undermined by the existing government jurisdictional system. The PRIME project has been searching for ways to support and strengthen its rangeland management roles and responsibilities.

In Garba Tula, the central government, through KWS, KFS, and NGAO make and implement decisions on natural resources that sometimes conflict with the dheeda council. At the same time, the dheeda council does not include elders from other ethnic communities who usually would not respect all the decisions by the council. Borana women, who by tradition do not directly participate in dheeda council processes, do have senior positions in the county government. A women’s representative from this group has strongly opposed some provisions of the proposed community natural resources management County Assembly Bill, which was intended to formally legitimize the rangeland management structures on grounds of not including women. As well, other ethnic communities were omitted in the committees that spearheaded the re-invigoration of the dheeda council but are well represented in the county and central governments. For example, although the Abba-herega (an elder in charge of wells) is from the Somali community, some Somalis feel that attempts to reinvigorate Borana customary law will exclude them from sharing the resources. Influential people from these ethnic backgrounds are opposing attempts by RAP and others to encourage the County to pass legislation that would give the resource governance system and local by-laws at Garba Tula legal force.

In the case of Il’Ngwesi, twice within 2015, the community experienced significant cases of incursion into the group ranch by herders from elsewhere determined to access pastures conserved by the community for its dry season grazing needs. During the field study, Samburu herders facing pasture and water shortages due to failed rains, invaded and settled in sections of land earmarked by Il’Ngwesi for later grazing. Meetings were held, but the Samburu, who were armed with firearms, defied directives to move out and continued grazing in Il’Ngwesi land. It took the collective effort of the national and county government security agencies to normalize the situation. In the case that we observed, government security agencies responded positively and helped eject the invaders, but not until the reserved dry season grazing areas had been devastated. Respondents from Il’Ngwesi complained that the governments protect the property rights of private ranches and game reserves in the area much more quickly and effectively than they protect the property rights of group ranches. We were unable to establish whether such complaints were accurate. However, even the subjective *impression* that the property rights of group ranches are second class property rights highlights that the challenges are not only in terms of horizontal relationships to other communities and the extent to which these other communities respect the legitimacy of the group ranch and its rights over its territory but also in terms of vertical relationships to government at county and national level and the extent to which they legitimize and enforce de jure governance powers of the group ranch.

## Discussion

In each of these three cases, there was a pre-existing, socially relevant landscape which afforded a geographic scope for the interventions. These pre-existing landscape definitions provided a history and logic for working at a scale that corresponded to the needs of pastoralist livelihoods. In all three cases, the strategy of the external agents involved a mix of both reinvigorating existing or historical elements of management and governance, but also creating new elements as necessary. In other words, in all three cases they seemed to be working at a scale and level that made sense, and in all three cases the external agents worked with local communities using a participatory approach. Moreover, each case has shown promising signs of community support for the landscape institutions and processes, and two of the three were in operation long enough to witness some success in managing pasture resources. In short, in all three cases the external agents seemed to work appropriately, and effectively at community and landscape levels.

Yet in all three cases, the organizations and institutions of governance for the landscape have been facing great difficulties in being able to exercise management authority. While there have been some difficulties in establishing the authority of the landscape institutions among the local community, these have been relatively minor. The challenges, rather, relate to the relationships beyond the landscape, both vertically to higher levels of decision-making and horizontally in relation to communities normally residing in other landscapes. The actual governance powers that the landscape institutions are able to exercise are insufficient to allow them to manage their resources, to exclude outsiders, or even to require outsiders to observe the same rules which local resource users follow, such as following seasonal grazing patterns.

As indicated earlier in the paper, commons scholarship has noted that local level commons should not be seen as discrete, self-contained “islands”; they are embedded within larger landscapes and exist within a multi-level world (Berkes 2009). However, landscapes too are embedded within larger watersheds, bioregions, and jurisdictions. Any attempt to foster or create effective governance at the landscape level must take into account the social and ecological environment within which the mechanisms of landscape governance are to function, and plan accordingly. In particular, the broader governance context is critical, because without its support, any mechanisms for management and governance at the landscape level may not be seen as legitimate by stakeholders from beyond that landscape. In the Gomole case, the government has never recognized the right of the rangeland council to make management decisions for the rangeland. Similarly at Isiolo County, attempts to formally legitimize the Garba Tula system of management at the County stalled. At Il’Ngwesi, the community’s acquisition of land beyond the group ranch in strategic mountain pasture zones indicates the need for and usefulness of nested multilevel resource management in maintaining sustainable pastoral livelihoods. However, residents of Il’Ngwesi experience routine challenges that pose threats to their resource governance system. While the tenure rights of group ranches are formally established, higher levels of government have been either unwilling or unable to consistently protect those rights when faced with an influx of livestock herds from other counties. In these cases, the NGOs supporting the communities have been much more effective at providing support at the local level within the landscape than they have been at playing the role of bridging organizations and at obtaining support for the communities from government at higher levels. Without the practical legitimization of the governance mechanisms for the landscape, these mechanisms do not achieve legitimacy in the eyes of other communities.

However, it is not only the institutional dimension that is important in these cross-level, cross-scale relationships. The relationship of the landscape to the broader social–ecological system is also important. While caution is needed in making generalizations from a small set of case studies, the findings of our research echo what others (e.g., Fernández-Giménez 2002; Moritz et al. 2013; Bollig and Lesorogol 2016) have observed about institutions in pastoralist systems: the nature of the physical environment has encouraged norms, institutions, and systems of management that differ from many other types of commons. In the three cases we have described, the variability of rainfall and forage, and the dominant livelihood strategy based on mobility, which this variability has compelled, are key. In pastoralist systems these characteristics have, over centuries, resulted in a conception of boundaries between territories being fuzzy and fluid. This is accompanied by an ethic which favours access over clearly defined ownership rights. These characteristics provide an impetus “from the bottom-up” against the strengthening or consolidation of governance powers, and against any neat resolution to what Fernández-Giménez (2002) calls the paradox of pastoral tenure.

**Fig. 1:**
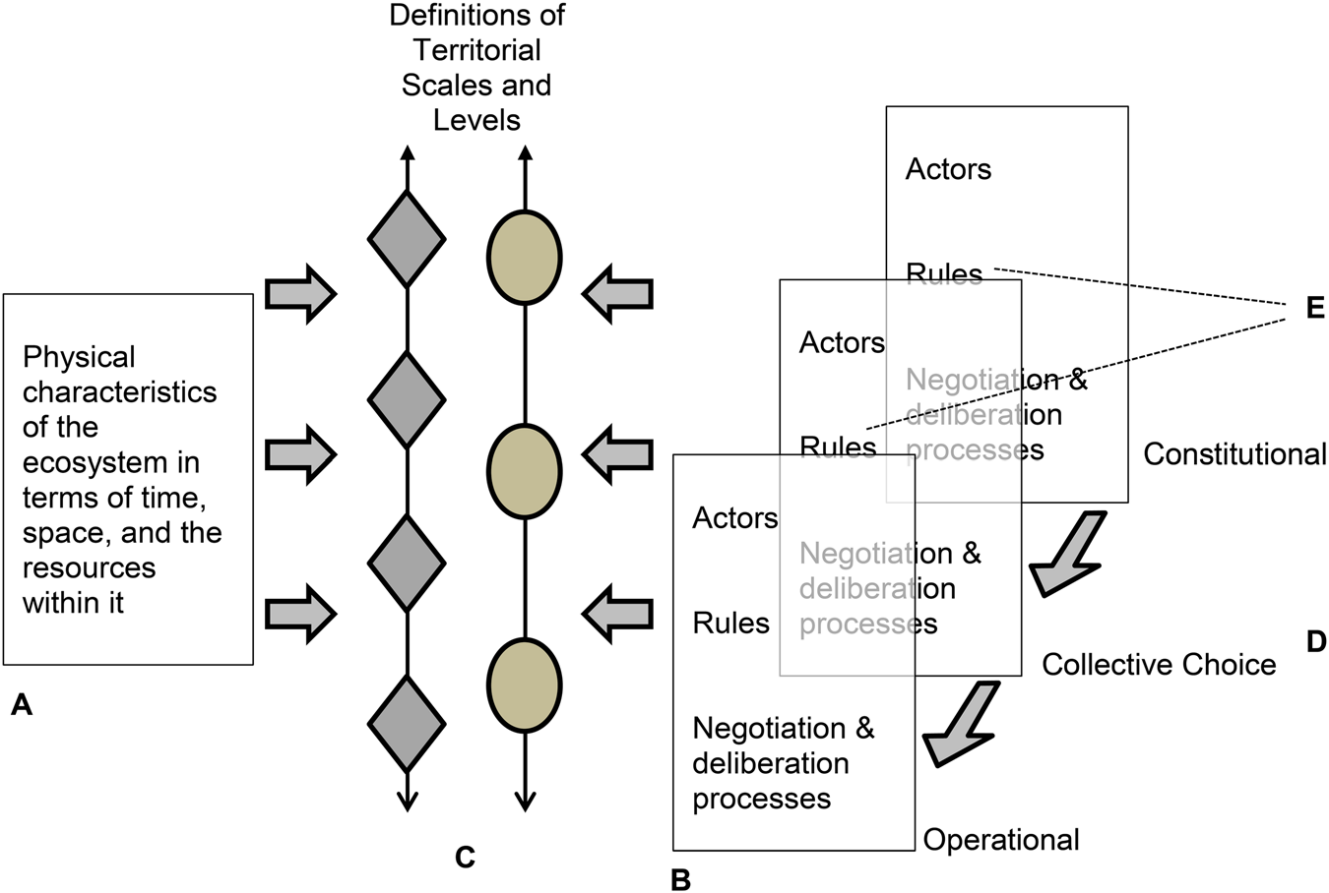
The materiality of multi-scale, multi-level commons. The definition of scales and levels is the result of the interaction between physical characteristics of ecosystems (**a**) and social elements involving various actors, rules, and negotiation and deliberation processes (**b**). The scales and levels making up a territory may be perceived differently by different actors (**c**). Rights to resources may be parsed according to timing of the right, methods of use, location and other characteristics. These rights may be allocated at different scales and levels through different sets of rules and different negotiation and deliberation processes to various different actors. Operational level decisions about allocation of and access to resources involve various actors, rules and negotiation and deliberation processes. These are shaped by actors, rules and negotiation and deliberation processes at collective choice level decisions, which are in turn shaped by constitutional level decisions (**d**). One of the common shortcomings preventing the emergence of functioning polycentric systems is the insufficiency of rules that can legitimize and enable community organization, negotiation and deliberation processes between actors, and the creation of operational rules (**e**)

These characteristics of dryland pastoralist rangelands have also resulted in complex configurations of scales and levels. The challenge is not simply the archetypical case of scale mismatch in which the jurisdictional scale does not correspond to the spatial social–ecological scale. Rather, there may be *multiple* jurisdictional scales: different communities, clans or ethnic groups may have decision-making systems that differ from each other and from the territories and corresponding institutions created by the state. There may also be different perceptions of social–ecological scales, and, with these, competing claims over resources—claims which differ in terms of the timing and spatial allocation of those rights and the rules that pertain. Maasai members of Il’Ngwesi group ranch, for example, see the group ranch as a unit collectively owned by the members with full and exclusive rights of management and exclusion of non-members, whereas Samburu pastoralists from beyond the landscape see themselves as having rights to graze in the area during droughts. These differing perceptions result in part from the spatio-temporal variability of the resources. Commons scholarship has noted that commons have a materiality such that they are the result of interacting physical and social factors (Bollig and Lesorogol 2016). What is underappreciated, however, is that the arrangement of commons in scales and levels and the ways the different actors define scales and levels are a fundamental part of how commons are organized, and are also the result of interacting physical and social factors. Physical characteristics of ecosystems that are relevant include elements of time, space, and the nature of resources (“A” in Fig. 1). In the systems described here, the spatial and temporal variability, and heterogeneity of pasture resources is a key driver of how scales and levels are perceived. Components of the social system which contribute to defining scales and levels in include actors (individuals, communities, and organizations), rules, and negotiation and deliberation processes (“B” in Fig. 1). Commons scholarship generally has devoted a great deal of attention to rules and much less to negotiation and deliberation processes, which traditionally have been very important in pastoralist governance systems.

Overlapping perceptions of territoriality and competing claims over resources do not in themselves preclude resolution. In the three situations described here, however, what is insufficient is a system of overarching collective choice and constitutional rules that would allow for functioning polycentricity (“E” in Fig. 1). The rights of communities to organize their own solutions is not sufficiently recognized or facilitated by existing rules. It is also important to recognize that the multiple decision-centers that characterize polycentric systems need not be only *organizations*. Traditional pastoralist decision-making is often based as much on negotiation and deliberation processes, and various sorts of traditional, ad hoc meetings as it is on organizations (Robinson et al. 2010). Allocating the rights to make certain operational, collective choice and constitutional decisions among various organizations, and negotiation and deliberation processes at various levels would help to create the enabling conditions for self-organization to occur. What we wish to emphasize is that decisions at operational, collective choice and constitutional levels result not only from nested rules, but from the interaction of actors, rules, and negotiation and deliberation processes (“D” in Fig. 1).

Recognition of the physical nature of resources, of the possibility for differing definitions of scales and levels, and of the interaction of actors, rules, and negotiation and deliberation processes operating across different scales and levels has implications for governance design. Rights to resources on the land do not need to be allocated on an all-or-nothing basis, with all rights to all resources on each patch of land allocated in perpetuity solely to a single actor. Instead, rights to resources can be parsed according to timing of the right, methods of use, and location. To offer a simple example, one local group may have exclusive access, use and management rights to certain pastures in normal rainfall years, while that same pasture is opened to more distant groups during drought years. The decisions about allocating these deconstructed rights to different patches of the resource can be allocated at different scales and levels to different actors, and to different negotiation and deliberation processes.

The lessons from these cases fall into two categories. The first relates to the need for government at higher levels to provide an enabling environment that supports and offers formal and practical legitimization of landscape-level governance mechanisms. Investing these governance mechanisms with stronger governance powers can help to avoid a situation of de facto open access. The findings from these three cases, however, also suggest that a caution is in order. Any attempt to create and strengthen landscape-level governance in a way that results in impermeable borders is likely to run up against the bottom-up impetus for flexibility and fluidity. Fostering effective landscape-level governance cannot be accomplished only through action at the landscape level; it is a task that must be pursued at multiple levels, and in relation to the connections across scales and levels.

While research on landscape governance has identified that effective landscape governance requires action not only at the landscape level but across scales and levels, the roles played by cross-scale and cross-level interactions in landscape governance remains poorly understood. The findings of these three case studies suggest that the characteristics of the physical environment in dryland pastoralist settings, the nature of the pastoralist livelihood based on mobility and opportunistic exploitation of resources, and the norms and institutions which have arisen in response to these characteristics are critical. These features of pastoralist landscapes are such that effective governance of rangelands cannot simply be a larger replication of local level commons. Government frameworks may help to legitimize the authority of landscape-level community-based institutions; we hypothesize, however, that the materiality of these commons means that rights of access, use, management, and exclusion will need to be nested across multiple levels and entail some degree of flexibility, as has been the case in many traditional pastoralist resource management systems. Rather than entrenching fixed and comprehensive management authority within a series of discrete, non-overlapping territories each with its well-defined membership, as suggested by Ostrom’s first design principle, fluidity, negotiation and overlapping rights are likely to be key features of effective landscape governance arrangements for pastoralists.

These are not characteristics common in governance regimes designed by modern states. Systems of landscape governance must be flexible enough that they can respond and adapt to the broader physical and social environment within which they are set. Scholarship on environmental governance and natural resources management has elaborated theory and identified examples that may be relevant. Folke and co-authors (2005) identified that adaptive governance is governance that is characterized by flexible institutional arrangements and multiple institutional and organizational linkages. Similarly, adaptive co-management is described (Kofinas 2009) as an approach in which intentional efforts are made across scales and levels, and in which constant monitoring, learning, and adaptation are part of the process. Rather than governance being based on discrete distributions of authority with each entity functioning in its own sphere, adaptive co-management is achieved through networks of decision-making arrangements with effective cross-level institutional linkages (Olsson et al. 2004; Armitage et al. 2007). One of the features of these kinds of adaptive systems that make them effective is the flexibility of institutional arrangements, and the organizational and institutional linkages. These provide a means for crafting governance systems, which can respond to the material conditions, and changes in the material conditions, of both the landscape level commons and the broader social–ecological systems within which they are set. Adaptive governance approaches, in other words, are able to take the materiality of different commons into account.

Implementing principles of adaptive governance and adaptive co-management in the design of governance systems in pastoralist settings will require organizations, institutions, and negotiation, and deliberation processes at various levels, including at the local/settlement, landscape/rangeland, large landscape/regional levels, as well as channels of communication between them, often facilitated by bridging organizations. Such a system will need to be sufficiently resourced, and recognized by governments and by both regular/local and occasional/distant resource users. An adaptive governance system in pastoralist settings will certainly mimic aspects of traditional pastoralist governance, with its reliance on deliberation and negotiation, but should also help to create an environment in which landscape level initiatives such as at Gomole, Garba Tula and Il’Ngwesi have sufficient security to incentivize both innovation and ongoing management of resources.

## Footnotes

1 See Graham et al. (2003) and Robinson and Makupa (2015) for a discussion of *governance powers*.
